# Recombinant Antigens Expressed in *Pichia pastoris* for the Diagnosis of Sleeping Sickness Caused by *Trypanosoma brucei gambiense*

**DOI:** 10.1371/journal.pntd.0003006

**Published:** 2014-07-17

**Authors:** Stijn Rogé, Liesbeth Van Nieuwenhove, Magali Meul, Annick Heykers, Annette Brouwer de Koning, Nicolas Bebronne, Yves Guisez, Philippe Büscher

**Affiliations:** 1 Department of Biomedical Sciences, Unit of Parasite Diagnostics, Institute of Tropical Medicine, Antwerp, Belgium; 2 Laboratory for Molecular Plant Physiology and Biotechnology, Department of Biology, University of Antwerp, Antwerp, Belgium; US Food and Drug Administration, United States of America

## Abstract

**Background:**

Screening tests for *gambiense* sleeping sickness, such as the CATT/*T. b. gambiense* and a recently developed lateral flow tests, are hitherto based on native variant surface glycoproteins (VSGs), namely LiTat 1.3 and LiTat 1.5, purified from highly virulent trypanosome strains grown in rodents.

**Methodology/Principal Findings:**

We have expressed SUMO (small ubiquitin-like modifier) fusion proteins of the immunogenic N-terminal part of these antigens in the yeast *Pichia pastoris*. The secreted recombinant proteins were affinity purified with yields up to 10 mg per liter cell culture.

**Conclusions/Significance:**

The diagnostic potential of each separate antigen and a mixture of both antigens was confirmed in ELISA on sera from 88 HAT patients and 74 endemic non-HAT controls. Replacement of native antigens in the screening tests for sleeping sickness by recombinant proteins will eliminate both the infection risk for the laboratory staff during antigen production and the need for laboratory animals. Upscaling production of recombinant antigens, e.g. in biofermentors, is straightforward thus leading to improved standardisation of antigen production and reduced production costs, which on their turn will increase the availability and affordability of the diagnostic tests needed for the elimination of *gambiense* HAT.

## Author Summary

Population screening for the chronic form of sleeping sickness or *gambiense* human African trypanosomiasis (HAT) is still based on an antibody detection test against the native variant surface glycoprotein (VSG) LiTat 1.3. This protein is produced through massive infections of lab animals with highly virulent parasites. We aim to replace this native antigen with recombinant VSGs, both LiTat 1.3 and LiTat 1.5, expressed in the yeast *Pichia pastoris*. The diagnostic potential of these recombinants was confirmed in ELISA with sera from HAT patients and negative controls. Replacement of the native LiTat 1.3 VSG with these recombinants would prevent the infection and sacrifice of lab animals and the inherent infection risk linked to the production of the screening test. Upscaling production of recombinant antigens, e.g. in biofermentors, is straightforward thus leading to improved standardisation of antigen production and reduced production costs, which on their turn will increase the availability and affordability of the diagnostic tests needed for the elimination of *gambiense* HAT.

## Introduction

African trypanosomiases are neglected tropical diseases that perpetuate poverty through their burden on both public health and agriculture [1]. Human African trypanosomiasis (HAT) or sleeping sickness occurs in remote sub-Saharan areas and is caused by two human infective subspecies of the protozoan parasite *Trypanosoma brucei (T.b.)*. *T.b. gambiense* is endemic in West and Central Africa, where it causes a chronic form of sleeping sickness. It is primarily considered as a human infection, but infections of domestic and wild animals might also be observed [2], [3]. The other human infective subspecies, *T.b. rhodesiense*, endemic in Eastern and Southern African countries, causes an acute form of sleeping sickness. It is a zoonosis with non-human vertebrates as primary reservoir [4]–[7]. The trypanosomes are transmitted by the bite of an infected tsetse fly (*Glossina* spp.) [8], [9]. Sleeping sickness can be cured, but early diagnosis is important since treatment of second stage patients is more complicated and the risk of severe side effects increases significantly [8].

In 2001, efforts to eliminate HAT were intensified. Since then the number of reported cases declined by more than 70% with 7214 new cases reported to the World Health Organisation (WHO) in 2012. Infected patients were only detected in 13 of the 24 historical *T.b. gambiense* endemic countries, with the vast majority (84%) in the Democratic Republic of the Congo. *T.b. rhodesiense* infections accounted for only 2% or 110 new cases in 6 countries; 65% of them in Uganda. The WHO envisages the elimination of *gambiense* HAT by 2030 through active and passive case detection combined with vector control [10].

The HAT control programs in *T.b. gambiense* endemic areas aim at parasite elimination from the human reservoir by means of mass screening, diagnosis and treatment of affected individuals. Accurate diagnosis of sleeping sickness not only prevents incorrect or delayed medical intervention possibly resulting in death of the patient, but also limits disease transmission in the community through a decrease of the human reservoir [11]–[14]. The diagnosis of *gambiense* sleeping sickness consists of three interrelated steps: screening, parasitological confirmation and staging [12], [15]. Currently the Card Agglutination Test for Trypanosomiasis (CATT), an antibody detection test, is used for mass population screening. Even though antibody detection techniques only provide indirect evidence for the presence of trypanosome infections, they are a valuable tool for mass screening because of the limited sensitivity of the parasitological tests [12], [16]. These parasitological tests are performed on lymph or blood and consist of microscopical parasite detection. The most sensitive diagnostic field test available is the mini-anion exchange centrifugation technique (mAECT); even more so when the trypanosomes are concentrated in the buffy coat [17], [18]. Staging of sleeping sickness is performed through microscopical examination of the cerebrospinal fluid for elevated white blood cells (more than 5 per µl) or for the presence of trypanosomes.

For diseases with often low parasitaemias, such as *gambiense* sleeping sickness, screening for the presence of specific antibodies elicited upon contact with the parasite, offers a valuable detection tool. The better screening tests for *gambiense* sleeping sickness are all based on variant surface glycoproteins (VSGs). One type of VSG covers the complete surface of the trypanosome, including the flagellum, by forming a dense layer of dimers. This VSG coat is highly immunogenic. The parasite can however avoid complete elimination by the host humoral immune system by regularly replacing the VSG coat by another one of a different antigenic type, a mechanism called antigenic variation [19], [20]. The CATT screening test for *T.b. gambiense* uses the LiTat 1.3 VSG as antigen [21]. This VSG is expressed early in most *gambiense* infections; therefore specific anti-LiTat 1.3 VSG antibodies serve as a potent diagnostic marker. To increase the sensitivity of the antibody detection test other predominant VSGs, LiTat 1.5 and LiTat 1.6, can be added. A rapid latex agglutination test, LATEX/*T.b. gambiense*, combining these three VSGs has been developed [22] and the ELISA/*T.b. gambiense* with the same antigen combination has a proven high sensitivity and specificity on serum, plasma, CSF and even saliva [23], [24]. However, stability and/or logistical requirement issues prevented replacement of the CATT by these tests. Recently, rapid lateral flow diagnostic tests for *gambiense* HAT have been developed which use a combination of native LiTat 1.3 and LiTat 1.5 VSGs as antigens [25]–[27]. Compared to the CATT, these tests are as sensitive and specific and fully comply with the ASSURED criteria defined for rapid diagnostic tests [28]. However, the native antigens for these tests are still produced through massive infections of laboratory rodents with highly human-infective bloodstream form trypanosomes expressing these variant antigenic types (VATs). We therefore aim to replace these native antigens by recombinant antigens in order to eliminate the infection risk for staff and the need for laboratory animals for antigen production. We opted for secreted expression of the expressed VSG fragments to avoid the difficult and tedious purification of intracellular, recombinant proteins. Since eukaryotic post-translational modifications, such as the glycosylation pattern of a protein, can be important for the correct folding of the protein and hence its diagnostic value, we expressed the antigens recombinantly in the yeast *Pichia pastoris*. We have previously used the same expression host to successfully express the *T. evansi* VSG RoTat 1.2 [29]. The expressed and secreted recombinant proteins were affinity purified and tested for their diagnostic potential with a panel of sera from HAT patients and non-HAT controls.

## Materials and Methods

### Ethics statement

Sera from HAT patients and endemic non-HAT controls were collected within different diagnostic studies [24], [30]. All individuals gave their written informed consent for the use of their plasma specimen in HAT research before providing blood. Permission for these studies was obtained from the national ethical committee of the Democratic Republic of the Congo (DR Congo) and from the Institute of Tropical Medicine Antwerp (ITMA) ethical committee, reference number 03 07 1 413 and 04 44 1 472. All specimens were anonymised.

### Yeast strain

For recombinant expression of the trypanosome proteins, the *Pichia* GlycoSwitch M5 strain was used to assure homogeneous, trypanosome-like Man_5_GlcNAc_2_ N-glycosylation of the secreted proteins [31].

### Expression vectors

The recombinants were cloned in the pP-αhSUMO3 vector of the SUMOpro-3 Gene Fusion Technology kit (LifeSensors) and electroporated in the *Pichia* GlycoSwitch M5 strain. This vector contains the strong, methanol-inducible AOX1 promoter followed by an alpha-mating factor signal sequence for secreted expression of the recombinants [32] and a N-terminal His tag for downstream affinity purification. The affinity tag is linked to the human SUMO3 (small ubiquitin-like modifier) which precedes the cloned protein-of-interest in order to enhance the expression and to promote the solubility and correct folding of its fusion partner.

The cloned constructs in the pP-αhSUMOpro3 vector result in SUMO fusion proteins. The rLiTat 1.3 consists of a N-terminal His tag, followed by the SUMO fusion protein, the 349 N-t amino acids of LiTat 1.3 VSG and a C-t Strep tag II. The rLiTat 1.5 has a similar setup, with 394 N-t amino acids of the LiTat 1.5 VSG.

### Preparation of cDNA from purified trypanosomes of *T.b. gambiense* LiTat 1.5

The trypanosomes expressing the LiTat 1.5 VSG were obtained from the collection of trypanosome stabilates maintained at the Institute of Tropical Medicine in Antwerp. Cryostabilates were injected in OF-1 mice. At the first peak parasitaemia, the mice were euthanised and bled by cardiac puncture. The trypanosomes were purified from the blood through ion exchange chromatography on DEAE cellulose according to Lanham & Godfrey [33]. The purified trypanosomes were centrifuged (15 min, 1500 g) and washed twice with 14 ml of phosphate buffered saline-glucose, pH 8.0, and then pelleted by centrifugation. The pellet was either used immediately or stored at −80°C for later use. Total RNA was extracted from the pellets with the RNeasy Midi Kit (Qiagen). Finally, the RNA was reverse transcribed with an oligo-dT primer to cDNA following the Omniscript RT Kit protocol (Qiagen).

### Construct engineering

The variable N-terminal parts of LiTat 1.3 and of LiTat 1.5 VSG were cloned in the expression host since they contain the specific immunogenic epitopes of the VSGs which are exposed to the host immune system. The N-terminal part of the LiTat 1.3 sequence (GenBank accession no. **KJ499460**) was PCR-amplified from *T.b. gambiense* LiTat 1.3 genomic DNA (gDNA) with a primer set starting at the first residue of the mature polypeptide (amino acid 24) and ending at amino acid 372 of a total of 479 amino acids. A *Bsa*I site was incorporated in the forward primer (LiTat 1.3_SUMO-FP) and a Strep tag II coding sequence (IBA), a stop codon and a *Bsa*I*/Xba*I site were added to the reverse primer (LiTat 1.3_SUMO-RP). The N-terminal domain of the LiTat 1.5 VSG sequence was amplified from *T.b. gambiense* LiTat 1.5 cDNA (GenBank accession no. **HQ662603**). These primers were developed according to the instructions of the cloning strategy (In-Fusion HD Cloning Kit, Clontech Laboratories). The complete LiTat 1.5 VSG sequence covers 502 amino acids, but the amplified sequence starts at the first residue of the mature polypeptide (amino acid 33) and ends at amino acid 426 [34]. All primers used are shown in table 1.

**Table 1:** PCR primers developed for the recombinant expression of LiTat 1.3 and LiTat 1.5 constructs with incorporation of a C-terminal Strep tag II.

LiTat 1.3_SUMO-FP	ATA	GGTCTCAAGGT	GCTGTCAATGGCAATGTC		
	Red	*Bsa*I	LiTat 1.3 **VSG AJ304413** (AA 24)		
LiTat 1.3_SUMO-RP	ATT	GGTCTCTCTAGA	TTA	CTTTTCAAATTGTGGATGAGACCA	GGCTAGCTGTGTGTCCTG
	Red	*Bsa*I*/Xba*I	Stop	Strep tag II	LiTat 1.3 **VSG AJ304413** (AA 372)
LiTat 1.5_SUMO_IF-FP	CAGCAGACGGGAGGT		GCG GCC ATA ACC GAT		
	pP-αSUMOpro3 vector		LiTat 1.5 **VSG HQ662603** (AA 33)		
LiTat 1.5_SUMO_IF-RP	CCGCGGCCGCTCTAGT		TTA	CTTTTCAAATTGTGGATGAGACCA	TGATGTTTCAATTTCTTTTTTG
	pP-αSUMOpro3 vector		Stop	Strep tag II	LiTat 1.5 **VSG HQ662603** (AA 426)

Red: redundant nucleotides, FP: forward primer, RP: reverse primer, IF: In-Fusion.

The PCR fragments were cloned in the pP-αhSUMO3 vector, previously digested with *Bsm*BI. The resulting constructs were transformed into TOP10 *E. coli* cells (Invitrogen) and plated on LB_LS-z_ (low salt Lysogeny Broth medium with 25 µg/ml zeocin (InvivoGen)) agar plates. Positive clones were selected by colony PCR with α-factor and 3′AOX1 primers and grown overnight in 5 ml LB_LS-z_ medium in a shaker incubator at 37°C and 250 rpm (Innova 44R, New Brunswick Scientific). The plasmid DNA was purified (QIAprep Spin Miniprep Kit, Qiagen) and the nucleotide sequences of the truncated genes in the transfer plasmid were confirmed by sequencing.

For the LiTat 1.3 construct a secreted rLiTat 1.3_H-SUMO-24-372-Strep_ (rLiTat 1.3) protein of 457 amino acids with a calculated mass of 49 kDa is expected, while the LiTat 1.5 construct codes for rLiTat 1.5_H-SUMO-33-426-Strep_ (rLiTat 1.5) consisting of 502 amino acids with a theoretical mass of 55 kDa (ExPASy ProtParam tool).

### Transfection into *Pichia pastoris*

The constructs were linearised with *Pme*I and purified (QIAquick, QIAGEN). The concentration was measured with the NanoDrop ND-1000 Spectrophotometer (ISOGEN Life Science) and 1 to 3 µg linearised plasmid DNA in a maximum volume of 10 µl were electroporated (25 µF, 2000 V; Gene Pulser XCell, Bio-Rad) into 80 µl electrocompetent *Pichia* GlycoSwitch M5 cells. The transfected clones were selected on YPDS_z_ (yeast extract peptone dextrose sorbitol medium with zeocin) agar plates with zeocin concentrations ranging from 100 to 2000 µg/ml to select for putative multicopy transfectants.

### Screening for expression of recombinant LiTat 1.3 and 1.5

Positive transfectants were selected by inoculation of individual colonies in 10 ml Buffered Glycerol-complex Medium (BMGY) in 50 ml falcon tubes. After 24 h growth (29°C, 250 rpm; Innova 44R, New Brunswick Scientific), the cells were collected by centrifugation for 5 min at 3220 g. The supernatant was discarded and the cells were resuspended in 10 ml Buffered Methanol-complex medium (BMMY).

The secreted expression of the recombinants was induced for 24 h at 250 rpm and, to minimise protein degradation, at 22°C. The supernatant was collected by centrifugation of the cell culture for 15 min at 3220 g. The proteins in 1 ml supernatant were precipitated with trichloroacetic acid (TCA) and resuspended in 25 µl of 1 M Tris-base and 25 µl of SDS-PAGE reducing loading buffer (0.125 M Tris-HCl (pH 6.8), 4% SDS, 20% sucrose, 0.04% bromophenol blue, 0.2 M dithiothreitol). The secreted proteins were separated over a 12% polyacrylamide gel (50 min at 200 V; Mini-PROTEAN Tetra Cell Electrophoresis System, Bio-Rad) and transferred to a nitrocellulose membrane by western blotting (30 min at 100 V; Criterion Blotter, Bio-Rad). The nitrocellulose membrane was blocked overnight with TBS-Blotto (0.02 M Tris-HCl (pH 7.5), 0.5 M NaCl, 0.004% NaN_3_, 5% skimmed milk powder (Fluka)) The proteins were visualised by immunostaining either indirectly with mouse anti-His tag antibody (1∶500; AbD Serotec) as primary antibodies followed by alkaline phosphatase conjugated rabbit anti-mouse IgG (1∶15000; Sigma) or directly with Strep-Tactin AP conjugate (1∶4000; IBA), followed by the addition of the substrates nitro blue tetrazolium (NBT) and 5-bromo-4-chloro-3-indolyl phosphate (BCIP).

### Bulk protein expression and purification

The selected protein secreting colonies were inoculated in 500 ml BMGY with addition of 0.2 g/l chloramphenicol. The cultures were grown in Fernbach shake flasks for 24 to 66 hours at 29°C and 200 rpm to create sufficient biomass. Afterwards, the cells were collected by centrifugation (5 min at 4417 g) and resuspended in an equal volume of BMMY_2%CA (BMMY supplemented with 2% casamino acids (BD)) containing 1% methanol and with addition of 1% J673A antifoam (Struktol) [35]. After measurement of the optical density at 600 nm the culture was further diluted with BMMY_2%CA to an OD_600 nm_ of 5.0 and 500 ml of this dilution was transferred to a Fernbach flask. Protein expression was induced for 2 days at 22°C and 200 rpm with addition of 0.5% methanol every 8 h. The supernatant was collected by centrifugation for 30 min at 17670 g (without brake).

The rLiTat 1.3 and 1.5 constructs were purified over a nickel charged nitrilotriacetic acid agarose resin (Ni-beads; HisPur Ni-NTA resin; Pierce). The collected culture supernatant was added to the Ni-beads (50 ml per 1 ml beads in a 50 ml falcon) and mixed for 1 h on a roller mixer at room temperature. After 1 h the beads were collected by 5 min centrifugation at 700 g. A sample of the supernatant (flow-through) was taken to evaluate the binding efficiency. The beads were resuspended in 1 ml of wash buffer (20 mM sodium phosphate, 300 mM sodium chloride (PBS) with 25 mM imidazole, pH 7.4) and decanted into a 20 ml Econo-column (Bio-Rad) in a cold room at 4°C. All further purification steps were performed in this cold room. After packing of the column, the unbound proteins were washed away with wash buffer until the baseline absorption value at 280 nm (i.e. the absorption value of the wash buffer), measured with an UV spectrophotometer (Spectra/Chrom Model 280 UV Detector, Spectrum Chromatography), was reached. The bound proteins were eluted with elution buffer (PBS with 250 mM imidazole, pH 7.4).

### Protein concentration

The purified proteins were desalted over a HiPrep 26/10 Desalting column (GE Healthcare) with PBS at 4°C according to the manufacturer's instructions. The concentration was measured by the BCA Protein Assay Kit (Thermo Scientific).

### Reactivity of the purified proteins in ELISA with human sera

Microplates (Maxisorp, Nunc) were coated overnight at 4°C with 100 µl/well of purified rLiTat 1.3 or rLiTat 1.5 at 4 µg/ml or a combination of both recombinants at 4+4 µg/ml and with 100 µl/well of native LiTat 1.3 or 1.5 at 2 µg/ml or a combination of both native antigens at 2+2 µg/ml. All antigens were diluted in PB (10 mM sodium phosphate, pH 6.5). Antigen-negative control wells were left empty. Further manipulations were done at ambient temperature. The protocol was based on the procedure according to Lejon *et al.* [36], [37]. To correct for aspecific reactions, caused by contaminating yeast proteins that were not eliminated from the protein mixture by the one-step affinity purification, a culture of untransfected *Pichia pastoris* M5 strain was grown for 47 h in BMMY_2%CA (after a pre-culture in BMGY). This culture was induced with methanol in exactly the same way as the transfected strains (i.e. addition of 0.5% methanol every 8 h). Before addition to the microplate the sera were desorbed by dilution in PBS-Blotto (0.01 M sodium phosphate, 0.2 M sodium chloride, 0.05% NaN_3_, 1% skimmed milk powder, pH 7.4) supplemented with 10% (v/v) untransfected M5 medium for 1 h at room temperature. The serum dilutions were centrifuged for 5 min at 3000 g before pipetting. One hundred and sixty two human sera from 88 *T.b. gambiense* HAT patients and 74 non-HAT controls, were tested at a 1∶400 dilution. Antibody binding was visualised with goat anti-human IgG (H+L) conjugated with horseradish peroxidase (1∶40000; Jackson ImmunoResearch) and the chromogen ABTS (2,2′-azinobis[3-ethylbenzothiazonline-6-sulfonic acid]-diammonium salt; Roche). The optical densities were read at 414 nm (Multiskan RC Version 6.0; Labsystems).

The optical density (OD) measured for each serum was recalculated as percent positivity (PP) of the OD of the strong positive control present on each plate. The same strong positive control and a weak positive control were incorporated in each plate to monitor plate to plate variations on a Levey-Jennings chart [38]. On this chart the Westgard rules were applied to accept or reject each individual plate and to repeat the ELISA if necessary [39].

### Statistical analysis

The accuracy of the different antigens for diagnosis was determined in SigmaPlot 12.0 by calculation of the area under the receiver operator characteristics (ROC) curve (AUC) [40]. Confidence intervals (CI) were determined according to DeLong [41]. Sensitivities and specificities with 95% binomial Wilson confidence intervals were calculated using SigmaPlot 12.0. The McNemar Chi2 test was used to test differences in sensitivity and specificity of the recombinant and native antigens and their mixtures and differences in AUCs.

## Results

### Expression and purification of rLiTat 1.3 and rLiTat 1.5

**Figure 1:**
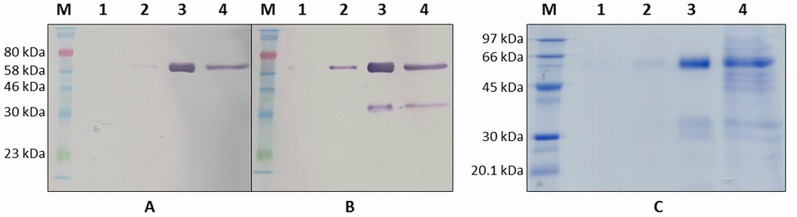
Expression of rLiTat 1. 3_H-SUMO-24-372-Strep_ by *Pichia pastoris*. A. Western blot with anti-His tag antibody; B: Western blot with anti-Strep tag antibody; C: Coomassie stained 12% SDS-PAGE gel; Protein markers (M): ColorPlus Prestained Protein Marker (NEB; A and B) and LMW-SDS protein marker (GE Healthcare; C); lane 1: supernatant of transfected *Pichia pastoris* M5 after 25 h induction; lane 2: supernatant of transfected *Pichia pastoris* M5 after 44 h induction; lane 3: Ni-NTA purified recombinant LiTat 1.3; lane 4: 20× concentrated flow-through of Ni-NTA purified supernatant.

After two days of induction, the secreted recombinants were affinity purified from the harvested supernatants and analysed by SDS-PAGE followed by western blot and Coomassie staining. The rLiTat 1.3 is detected in western blot with anti-His tag and anti-Strep tag II antibodies (Fig. 1, panel A and B). The anti-His tag antibody recognises a protein of approximately 60 kDa in the affinity purified fraction and in the 20× concentrated fraction that was not retained by the Ni-NTA resin. The anti-Strep tag antibody recognises the same 60 kDa protein in the supernatant of the *Pichia* culture after 44 h induction but also visualises a shorter, putative degradation product of approximately 35 kDa in the affinity purified fraction and the concentrated flow-through. Three bands are visible in the purified fraction in the Coomassie stained gel (Fig. 1, panel C). The most prominent band corresponds with the His- and Strep-tagged recombinant protein of 60 kDa. One of the two smaller bands of 30–35 kDa corresponds with the Strep-tagged degradation product of the recombinant protein. The apparent mass of 60 kDa of the rLiTat 1.3 does not match its expected theoretical mass of 49 kDa. However, the difference can be attributed to the SUMO fusion protein, since it is known that while this chaperone has a theoretical mass of less than 11 kDa, it migrates at approximately 20 kDa (Christian Loch, LifeSensors, personal communication). Not all secreted recombinant fractions were retained by the Ni-NTA resin since they are visible in the 20× concentrated flow-through of the Ni-NTA resin.

**Figure 2:**
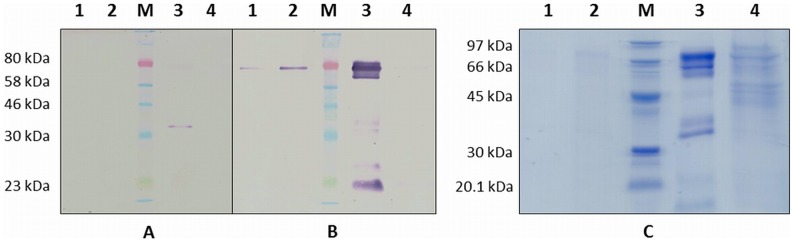
Expression of rLiTat 1. 5_H-SUMO-33-426-Strep_ by *Pichia pastoris*. A. Western blot with anti-His tag antibody; B: Western blot with anti-Strep tag antibody; C: Coomassie stained 12% SDS-PAGE gel; Protein markers (M): ColorPlus Prestained Protein Marker (NEB; A and B) and LMW-SDS protein marker (GE Healthcare; C); lane 1: supernatant of transfected *Pichia pastoris* M5 after 25 h induction; lane 2: supernatant of transfected *Pichia pastoris* M5 after 44 h induction; lane 3: Ni-NTA purified recombinant LiTat 1.5; lane 4: 20× concentrated flow-through of Ni-NTA purified supernatant.

The secreted rLiTat 1.5 also reacts with anti-His tag and anti-Strep tag II antibodies (Fig. 2, panel A and B). Only in the affinity purified fraction, the anti-His tag antibody recognises proteins of approximately 80 kDa (faint band) and 35 kDa. The anti-Strep tag antibody reacts already after 25 h induction with a 80 kDa protein in the supernatant of the *Pichia* culture and in the affinity purified fraction. In the latter fraction several putative degradation products of about 20, 25, 35, 40 and 70 kDa also react with this anti-Strep tag antibody. The apparent mass of the secreted recombinant is again higher than expected (80 kDa vs. 55 kDa). Moreover, this difference cannot be explained by the presence of the SUMO fusion partner alone. Nevertheless, we have previously observed that other recombinantly expressed trypanosome glycoproteins tend to migrate slower, probably due to interference of the oligosaccharides with the binding of SDS on the protein backbone [29], [42]. Most secreted recombinant fractions were retained by the Ni-NTA resin since they are not or hardly visible in the 20× concentrated flow-through of the Ni-NTA resin (Fig. 2, panel C).

Affinity purification of 100 ml yeast culture after two days of induction yielded typically 1 mg of recombinant proteins.

### Diagnostic potential for antibody detection in sleeping sickness patients

**Figure 3:**
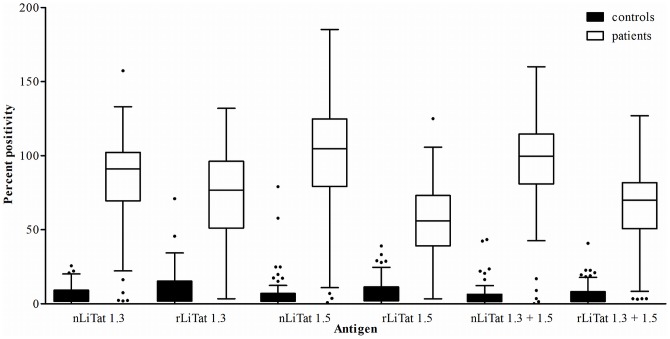
Box blots of the percent positivity (PP) obtained in ELISA with respectively native LiTat 1.3 (2 µg/ml), rLiTat 1.3_H-SUMO-24-372-Strep_ (4 µg/ml), native LiTat 1.5 (2 µg/ml), rLiTat 1.5_H-SUMO-33-426-Strep_ (4 µg/ml) and mixtures of both native (1+1 µg/ml) and recombinant (2+2 µg/ml) antigens tested with sera from 88 *T.b. gambiense* HAT patients and 74 non-HAT controls.

The diagnostic potential of rLiTat 1.3 and rLiTat 1.5 in comparison with their corresponding native antigens was assessed in ELISA with sera from 88 *T.b. gambiense* HAT patients and 74 non-HAT controls (Fig. 3 and Table 2). Both the recombinant and the native antigens reacted more strongly with the patient sera (10 to 34 times higher median PP values) than with the control sera. However, for each recombinant the median PP obtained with the patient sera was lower than for the corresponding native antigen with the same sera. A similar pattern was observed with mixtures of the two recombinant and of the two native antigens.

**Table 2:** Median percent positivity (PP) with 25^th^ and 75^th^ percentile obtained in ELISA with the different antigens and sera from 88 *T.b. gambiense* HAT patients and 74 non-HAT controls.

	Antigen	Median PP	25^th^ percentile	75^th^ percentile
Patients	nLiTat 1.3	91.0	69.6	102.2
	rLiTat 1.3	76.6	51.1	96.2
	nLiTat 1.5	104.8	79.2	124.9
	rLiTat 1.5	56.1	39.1	73.1
	nLiTat 1.3+1.5	99.6	80.9	114.7
	rLiTat 1.3+1.5	70.5	50.7	81.8
Controls	nLiTat 1.3	4.3	1.7	9.2
	rLiTat 1.3	4.5	1.8	15.2
	nLiTat 1.5	3.5	1.7	6.9
	rLiTat 1.5	5.4	2.0	11.4
	nLiTat 1.3+1.5	2.9	1.6	6.4
	rLiTat 1.3+1.5	3.4	1.5	8.1

**Figure 4:**
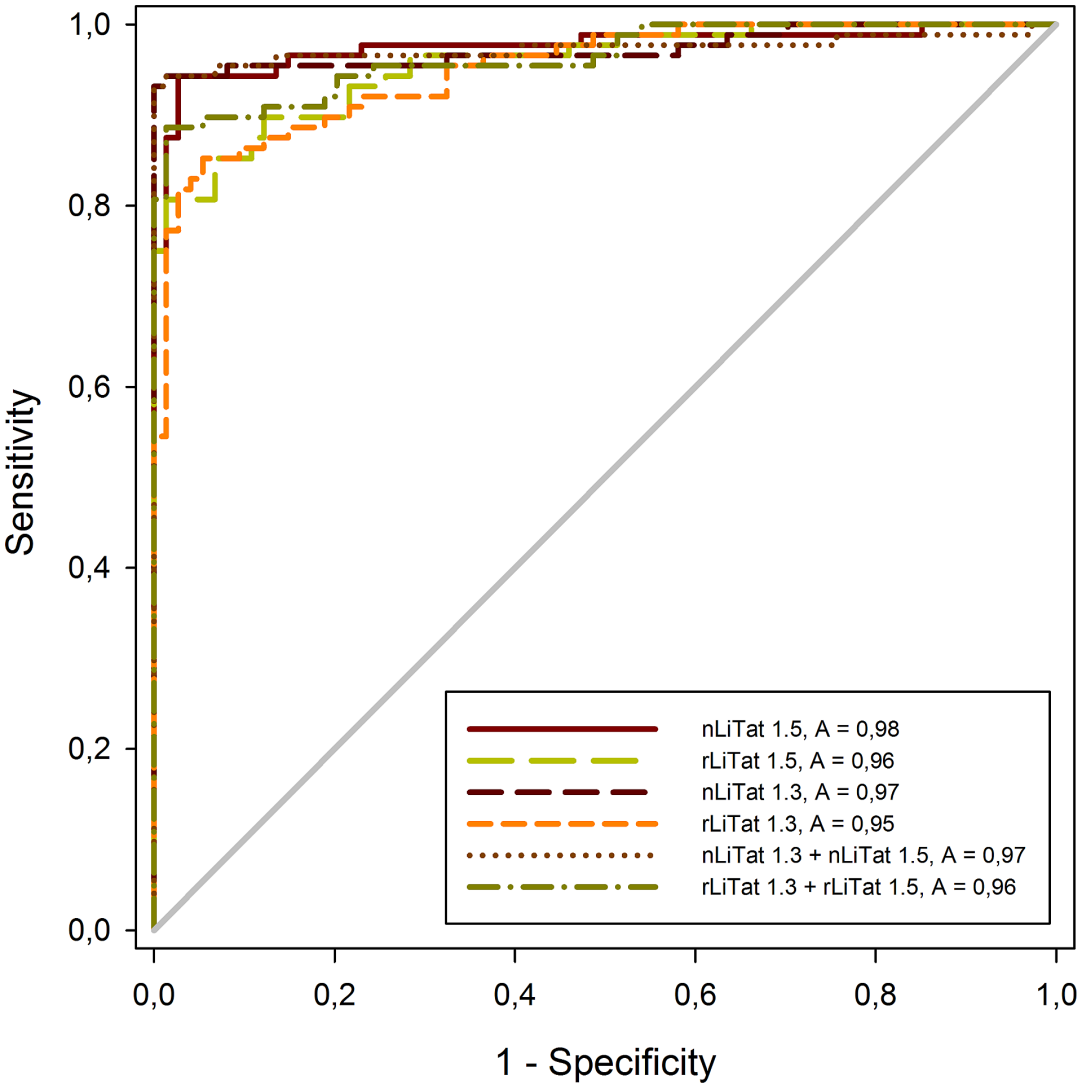
Receiver operator characteristic (ROC) curves and area under the curve (AUC) constructed from ELISA results obtained with native LiTat 1.3 (2 µg/ml), rLiTat 1.3_H-SUMO-24-372-Strep_ (4 µg/ml), native LiTat 1.5 (2 µg/ml), rLiTat 1.5_H-SUMO-33-426-Strep_ (4 µg/ml) and mixtures of both native (1+1 µg/ml) and recombinant (2+2 µg/ml) antigens tested with sera from 88 *T.b. gambiense* HAT patients and 74 non-HAT controls.

The relationship between diagnostic sensitivity and specificity of the antigens is represented as ROC curves in Figure 4. The area under the curve (AUC) is 0.97 (95% CI 0.948–0.999) for native LiTat 1.3 and 0.95 (95% CI 0.921–0.980) for rLiTat 1.3 and 0.98 (95% CI 0.952–0.999) for native LiTat 1.5 and 0.96 (95% CI 0.928–0.984) for rLiTat 1.5. With the mixture of the two native antigens an AUC of 0.97 (95% CI 0.944–1.000) is obtained while with the mixture of the two recombinant antigens, the AUC of 0.96 is slightly lower (95% CI 0.938–0.990). Pairwise comparison of the AUC for all antigens and their mixtures showed no significant differences (p>0.05).

For the whole range of cut-offs the Youden index was calculated (Youden index = sensitivity+specificity−1) [43]. For the cut-off PP value with maximal Youden index, the diagnostic sensitivity and specificity for each antigen and the combinations are represented in Table 3. With the individual recombinant antigens, the sensitivities are significantly lower than with the corresponding native antigens (p = 0.0027 for rLiTat 1.5 and p = 0.0196 for rLiTat 1.3). When both antigens are mixed, the sensitivity increases for the recombinant antigens but not for the native antigens and the difference becomes not significant (p = 0.1025). The specificity of recombinant LiTat 1.3 antigen is significantly lower than for its corresponding native antigen (p = 0.0455) while the specificities of recombinant LiTat 1.5 and the mixture of rLiTat 1.3 and rLiTat 1.5 are not significantly different from the specificities of the native LiTat 1.5 and the mixture of nLiTat 1.3 and nLiTat 1.5 (respectively p = 0.5637 and p = 0.3171).

**Table 3:** Percent positivity cut-off (max. Youden index) and sensitivity and specificity with 95% confidence intervals of the different antigens tested in ELISA with sera from 88 *T.b. gambiense* HAT patients and 74 non-HAT controls.

Antigen	Cut-off	Sensitivity	95% C.I.	Specificity	95% C.I.
nLiTat 1.3	29.6	93.2*	85.8–97.5	100.0*	95.1–100.0
rLiTat 1.3	30.3	85.2	76.1–91.9	94.69	86.7–98.5
nLiTat 1.5	26.0	94.3*	87.2–98.1	97.3	90.6–99.7
rLiTat 1.5	34.2	80.7	70.9–88.3	98.7	92.7–100.0
nLiTat 1.3+1.5	45.5	93.2	85.8–97.5	100.0	95.1–100.0
rLiTat 1.3+1.5	23.1	88.6	80.1–94.4	98.7	92.7–100.0

* significant difference between recombinant and native.

## Discussion

The variable N-terminal domains of two predominant *T.b. gambiense* VSGs, LiTat 1.3 and LiTat 1.5, were successfully expressed as SUMO fusion proteins by the yeast *Pichia pastoris* and secreted in the culture supernatant. The SUMO part prevents misfolding of its fusion partner and chaperones it through the endoplasmic reticulum [44]. In order to mimic the native antigen structure as much as possible, our recombinants were expressed in the GlycoSwitch M5 strain, ensuring a trypanosome-like Man_5_GlcNAc_2_ N-glycosylation profile.

The mature rLiTat 1.3 consists of 457 amino acids with two putative N-glycosylation sites on the expressed variable N-terminal domain of LiTat 1.3 VSG, namely Asn145 and Asn271 or amino acids 45 and 171 of the VSG. The mature rLiTat 1.5 consists of 502 amino acids with no putative N-glycosylation sites (NetNGlyc; ExPASy). However, in the absence of putative N-glycosylation sites in the rLiTat 1.5 fusion protein, O-glycosylation, which also occurs in *Pichia*, might account for its slower migration on SDS-PAGE [45]. As shown in the western blots and in the Coomassie stained gels secretion of the recombinants is accompanied by a certain level of degradation. During the optimisation phase of the expression protocol, we attempted to control degradation of the expressed protein by addition of several protease inhibitor preparations, however without success (data not shown). Neither did the addition of antifoam to the culture medium inhibit protein degradation although this resulted in a higher production. The combination of supplementation of the culture medium with antifoam and limiting the induction phase to maximum two days at 22°C provided the best means to control degradation of the expressed proteins with yields of up to 10 mg affinity purified recombinant protein per litre cell culture.

Another strategy that still could be attempted to avoid protein degradation, is the identification of the protease-sensitive sites through Edman degradation of the smaller fragments, followed by site-directed mutagenesis to change the identified protease recognition sequences.

Alternatively, the recombinant antigens could be expressed in *Pichia* strains that are deficient in vacuolar proteases, such as SMD1163, SMD1165 or SMD1168, in order to reduce protein degradation. However, these strains typically exhibit slower growth rates, lower transformation efficiencies and lower viabilities. Moreover, they are not available in the GlycoSwitch format [46].

Purification of the secreted recombinants was limited to a one step affinity purification on Ni-NTA resin. Additional purification via the C-terminal Strep tag II is possible but the diagnostic specificity of the recombinant proteins does not suggest that further purification is necessary. Finally, the SUMO fragment could also be removed from the fusion proteins with SUMO protease 2 but neither the sensitivity nor the specificity of the recombinants seem to be impaired by the presence of the SUMO protein.

The rLiTat 1.3 and rLiTat 1.5 antigens proved their diagnostic potential when compared to the corresponding native antigens in ELISA on sera from 88 HAT patients and 74 non-HAT controls. The slightly lower reactivity of the individual recombinants might be due to the fact they represent only the N-terminal fragments of the native VSGs, thus carrying less epitopes than their native counterparts. Furthermore, incomplete or incorrect dimerisation of the recombinants may impede the formation of certain diagnostically important conformational epitopes. These antigens are therefore ready to be incorporated in new rapid diagnostic tests. As mentioned in the introduction, new immunochromatographic antibody detection tests have been developed using the native LiTat 1.3 and LiTat 1.5 as antigens. This study proves that the rLiTat 1.3 and rLiTat 1.5, especially in combination, could replace the native antigens in a similar test format. Replacing the native antigens will eliminate infecting and sacrificing laboratory rodents and the inherent manipulation-related infection risk for the laboratory staff that is linked to the production of native antigens. Upscaling production of recombinant antigens, e.g. in biofermentors, is straightforward thus leading to improved standardisation of antigen production and reduced production costs, which on their turn will increase the availability and affordability of the diagnostic tests needed for the elimination of *gambiense* HAT.
